# Minimum requirements for a glaucoma programme

**Published:** 2022-01-31

**Authors:** Fatima Kyari, Rohit C Khanna

**Affiliations:** 1 Associate Professor: International Centre for Eye Health, London School of Hygiene & Tropical Medicine, UK. Consultant Ophthalmologist: College of Health Sciences, University of Abuja, Nigeria.; 2 Network Director: Allen Foster Eye Health Research Centre & Brien Holden Eye Centre, Gullapalli Pratibha Rao International Centre for Advancement of Rural Eye care, L V Prasad Eye Institute, Hyderabad, India.

**A successful glaucoma health care programme must provide a timely diagnosis as well as life-long monitoring and treatment of glaucoma.**

**Figure:**
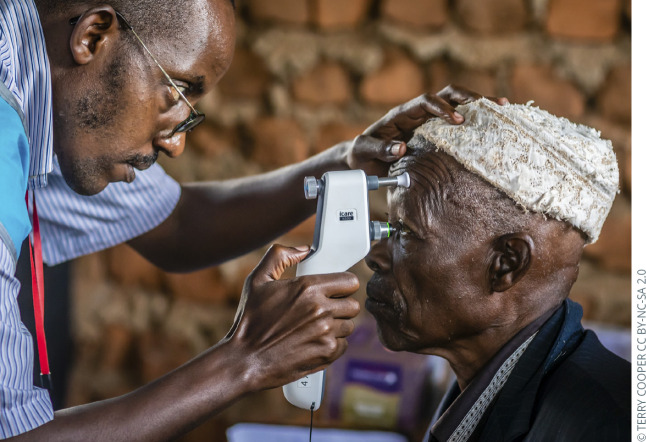
Screening for glaucoma; small, portable and easy-to-use devices allow screening of all at-risk patients at outreach clinics. **UGANDA**

**Figure 1:**
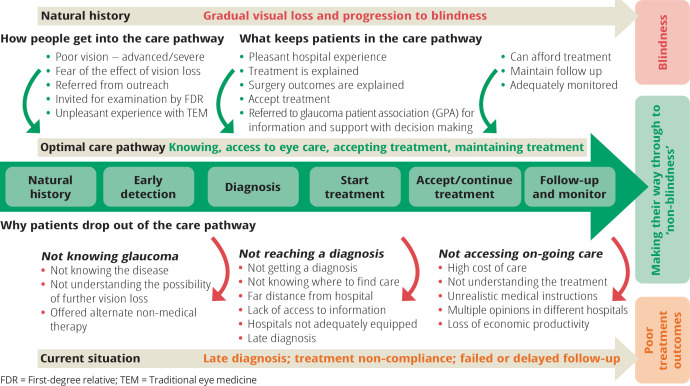
A conceptual framework for the glaucoma care pathway^1^

Glaucoma is a chronic non- communicable disease. A glaucoma care service can be most effective when it considers every aspect of the care pathway: from educating the patient who did not even know about glaucoma, to the patient receiving treatment and then maintaining compliance with treatment. The authors have previously developed a conceptual framework for the glaucoma care pathway (Figure 1). The conceptual framework for an optimal glaucoma care pathway considers engaging patients in glaucoma care from the community to the hospital and imagines that patients would take certain steps to avoid blindness.^1^ It takes into consideration important details about patients’ experiences such as awareness, uptake of health care services, and engaging with their glaucoma care. Understanding the patient’s journey will aid developing improved patient interaction processes that help promote earlier diagnosis as well as uptake of, and compliance with, treatment for glaucoma – with the aim of preventing vision loss and blindness from the disease.

The six stages needed for good glaucoma service delivery are:

1. Raising awareness
2. Access to care and earlier detection of glaucoma
3. Reaching/making a diagnosis
4. Accepting and choosing treatment
5. Compliance with treatment
6. Follow-up and monitoring to detect and treat disease progression.

All of these must be part of an integrated eye care set-up and not delivered in isolation.

There is generally poor awareness of glaucoma as a potentially blinding eye condition. **Raising awareness** among the general public involves a sustained effort to educate the public and boost their knowledge about glaucoma. We suggest providing targeted and appropriate messages via:

- Mass media (e.g., radio, TV, newspapers, information leaflets, magazines, etc.)
- Social media
- Health promotional activities, such as screening for non-communicable diseases
- Special awareness sessions during global events such as World Glaucoma Week or World Sight Day
- Interactive forums, both online and in the community, where patients can learn about their condition and ask questions.

With better health education and awareness, patients can develop the confidence to accept and adhere to treatment.

To improve patients’ **access to care**, community and primary health care workers can be trained to identify patients with, or at risk of, glaucoma. They should also be involved in increasing awareness among patients and monitoring their compliance with treatment and follow-up. Patients at risk include older adults, first-degree relatives of patients with glaucoma, and people with high intraocular pressure (IOP). It is useful to have a family screening database (or software) to monitor first-degree relatives.

At primary level, a technician can be trained to screen for glaucoma. At the L V Prasad Eye Institute in India, at primary or vision centre level, the Van Herrick test – along with applanation tonometry readings and non-mydriatic images of optic discs – are transmitted to the telemedicine centre for glaucoma detection. Additionally, follow-up care and compliance with therapy are monitored at this level.

The **toolkit for glaucoma management in Africa**^2^ describes the glaucoma care team according to competencies. Depending on their level of competency, members of the eye care team should be able to identify those at risk of vision loss due to glaucoma, provide care for patients with diagnosed and stable glaucoma, initiate treatment, and continue appropriate care for patients with glaucoma in order to prevent vision loss.

Other approaches to earlier detection include systematic population screening and opportunistic case-finding.

Secondary and tertiary eye care facilities should be adequately strengthened to enable them to **reach a diagnosis** of glaucoma. This requires appropriate equipment, trained, skilled personnel, and good information and management systems. Basic examination includes assessment of the optic nerve head, indentation gonioscopy for anterior chamber angle examination, measurement of central corneal thickness, visual field assessment, and optic disc imaging.

For details of equipment – refer to the **IAPB essential list for glaucoma** (**bit.ly/IAPBglauc**) which also categorises the equipment as ‘essential’ or ‘desirable.’

**Essential equipment**

1. For basic examination: torch, visual acuity charts, ophthalmoscope, tonometer
2. For specialist/diagnostic examination: slit lamp, slit lamp lenses (e.g., +90D, +78D), applanation tonometer, gonioscopy lenses, pachymetry, and visual fields.
3. Diagnostic pharmaceuticals: anaesthetic eye drops, fluorescein, dilating eye drops, sodium hypochlorite solution for disinfection (and sterile saline as rinse), and coupling gel (e.g., methylcellulose)
4. Glaucoma surgical instrument set, surgical pharmaceuticals and supplies.
5. IOP-lowering medicines.

Once a diagnosis of glaucoma is made, the patient and care provider are encouraged to ensure that **treatment** starts as soon as possible and continues for as long as needed. The choice of treatment should be based on the risk that a patient’s vision loss will progress. Consider the following:

- Stage of disease
- Sociodemographic and economic profile of the patient
- Family history of glaucoma or vision loss
- Systemic and ocular co-morbidity.

To enable **maintaining treatment** and keeping the patient in the care system, the following are important:

- **Service responsiveness.** This includes a pleasant hospital experience.
- **Counselling.** Inform the patient about the natural history of the disease, the irreversible vision loss it causes, the available interventions and purpose of treatment, and the need for long-term follow-up (including hospital visits). Cost of care and affordability, opportunity costs and loss of economic productivity should also be discussed.
- **Patient participation in their care.** The knowledge shared during counselling empowers patients to choose the most appropriate treatment through a shared decision process with the health care provider. Patient forums are also useful to encourage patients’ representation and contribute to how they engage in care.^3^ For example, through glaucoma patients’ groups and feedback, they can discuss individual concerns and suggest how the clinic/counselling spaces are organised. Patient groups may also provide peer support, e.g. patients can talk about obtaining medicines and taking them.

**“To improve patients’ access to care, community and primary health care workers are to be trained to identify patients with, or at risk of, glaucoma.”**

**Follow-up** care is required for **monitoring** and optimising treatment in response to the progression of the disease. Active mechanisms for contacting patients for follow-up include the use of:

- Clear follow-up instructions and provision of appointment dates
- Keeping patients contact details for texting/calling for reminders
- Glaucoma ambassadors – volunteers within the community who encourage patients in their own care.
